# Isolated Malignant Pleural Effusion in a Child: Unusual Presentation of Acute Leukemia

**DOI:** 10.7759/cureus.54232

**Published:** 2024-02-15

**Authors:** Srinija Garlapati, Sampada Tambolkar, Sarita Verma, Nakul V Pathak, Manojkumar G Patil

**Affiliations:** 1 Pediatrics, Dr. D. Y. Patil Medical College, Hospital & Research Centre, Dr. D. Y. Patil Vidyapeeth (Deemed to be University), Pune, IND; 2 Pediatric Oncology, KEM Hospital Research Centre, Pune, IND

**Keywords:** pleural fluid analysis, askins tumor, hematolymphoid malignancy, mediastinal mass, pleural effusion

## Abstract

Pleural effusion in the pediatric population is an abnormal pathology characterized by the accumulation of fluids between the parietal and visceral pleura. The etiology of this excessive fluid accumulation can be attributed to both infectious and non-infectious factors. Notably, *Streptococcus pneumoniae* stands out as the predominant infectious agent responsible for this condition. Non-infectious causative factors encompass hematolymphoid malignancies, congestive heart failure, hemothorax, hypoalbuminemia, and iatrogenic causes. Among the hematolymphoid malignancies, lymphoma emerges as the most prevalent malignancy associated with pleural effusion. It is followed by T-cell lymphoblastic leukemia, germ cell tumor, neurogenic tumor, chest wall and pulmonary malignancy, carcinoid tumor, pleuro-pulmonary blastoma, and Askin's tumor, among others. Malignant pleural effusion is predominantly linked to T-cell lymphoblastic malignancies. In the context of acute lymphoblastic leukemia (ALL), cases where T-cell presentation is accompanied by leukemic pleural effusion are commonly associated with either a mediastinal mass or significant lymphadenopathy. Here, we describe a case of a four-year-old male child who exhibited a brief history of febrile illness. Notably, this case was characterized by isolated pleural effusion, devoid of any mediastinal mass or lymphadenopathy. Pathological investigations of pleural fluid analysis revealed the presence of malignant cells, facilitating an expedited diagnosis.

## Introduction

Pleural effusion is a common condition, with an overall incidence of 0.32% in developed nations [1]. Research focusing on the causes of pleural effusion in children indicates that approximately half of cases are attributed to pneumonia, with malignancy, renal diseases, and trauma being other significant contributors. Bacterial infections are the primary cause of infectious pleural effusion, posing the risk of complications, such as empyema. However, viral infections, often asymptomatic, can also be associated with effusion. Pulmonary tuberculosis is reported in a wide range of cases, from 2% to 38% [2].

In the pediatric population, malignancy emerges as an important etiological factor for pleural effusion. Lymphoma, specifically T-cell lymphoblastic leukemia, is the most prevalent malignancy linked to pleural effusion, followed by germ cell tumor, neurogenic tumor, chest wall and pulmonary malignancy, carcinoid tumor, pleuro-pulmonary blastoma, and Askin's tumor. Lymphoblastic leukemia is the most common malignancy in pediatric age groups, typically presenting with fever, pancytopenia, lymphadenopathy, hepatosplenomegaly, and bone pain [3].

## Case presentation

A four-year-old male child, born out of a non-consanguineous marriage with no significant perinatal history and normal development, presented to the emergency department with a history of dry cough with no diurnal variation and not resolving with symptomatic management for 15 days. The patient also experienced shortness of breath for the last three days and pain in the abdomen for one day. One month back, the child had experienced a low-grade fever that relieved in one day on paracetamol. Computed tomography (CT) thorax done externally on the day of admission showed moderate right-sided loculated pleural effusion with complete collapse of the right lower lobe. On examination, the patient was found to have moderate dehydration and was also in respiratory distress. The vital signs indicated normal temperature, heart rate of 126 bpm, respiratory rate of 50 breaths per minute, and blood pressure of 112/68 mmHg. Oxygen saturation was found to be 86% on room air and 96% on continuous positive airway pressure (CPAP) at 5 liters per minute. The patient exhibited a mildly congested throat, with no evident lymphadenopathy or clubbing. Notably, the growth parameters were within the normal range. Examination of the chest showed asymmetry in chest expansion, characterized by a reduction in air entry on the right side, and bronchial breathing was also not auscultated. In addition, dullness on percussion was noted on the right side. Other systemic examinations did not reveal any notable findings.

**Figure 1:**
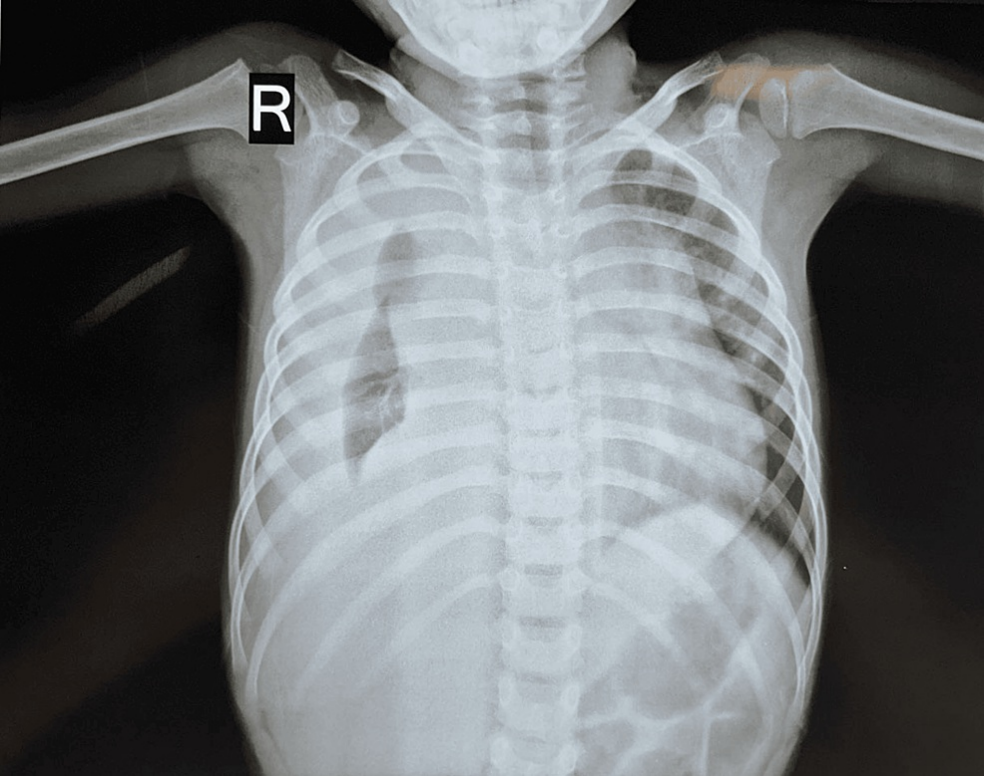
Initial chest X-ray showing diffused opacity

**Figure 2:**
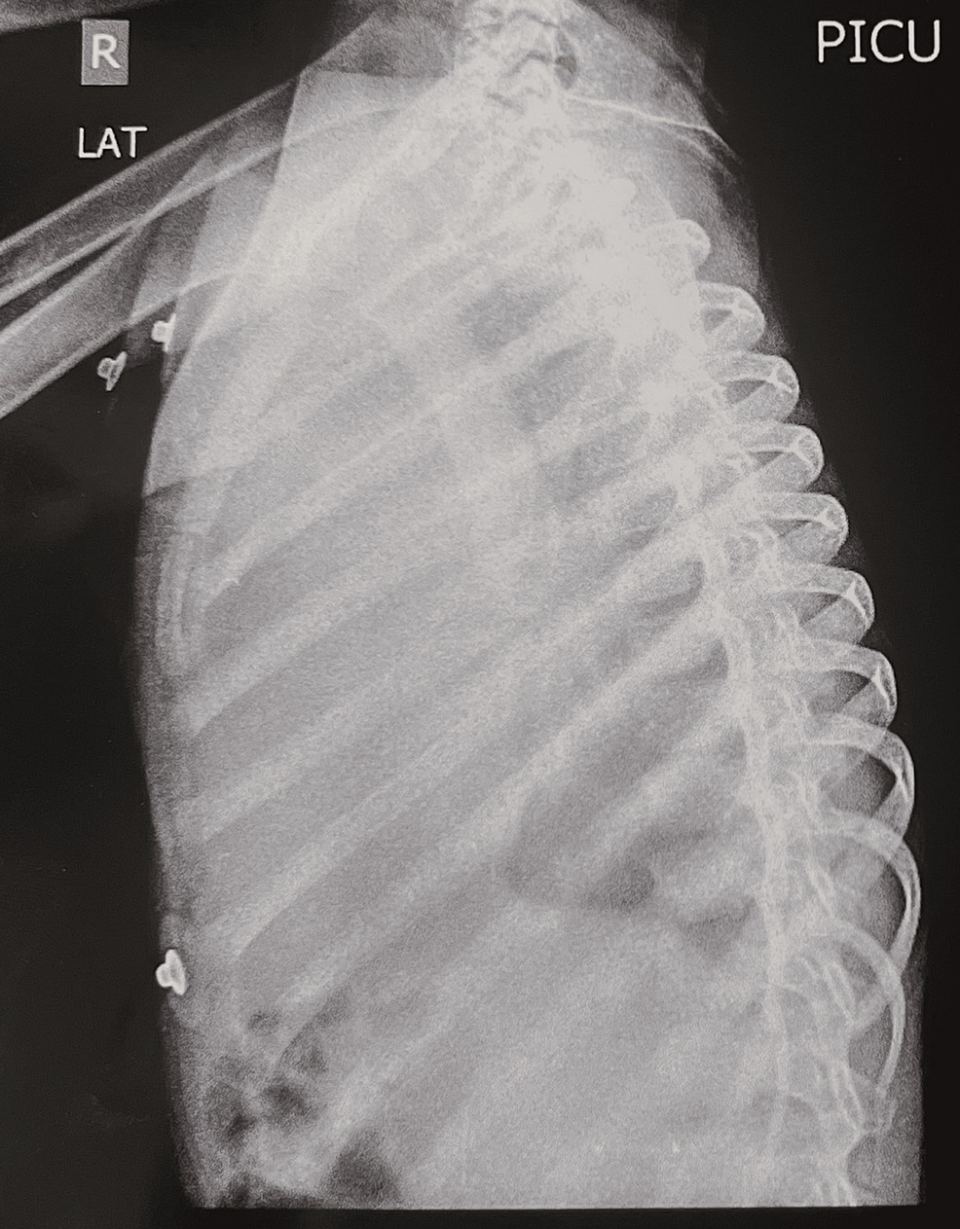
Plain chest X-ray in the right lateral decubitus view

The initial X-ray of the chest (Figure 1) revealed a diffused opacity predominantly on the right side of the chest, obscuring the costophrenic and cardiophrenic angles. Deviation in the trachea toward the left side and absence of an air bronchogram were observed. Furthermore, the right lateral decubitus positioned in plain chest X-ray revealed a substantial free fluid level, which occupied more than half of the ipsilateral hemithorax (Figure 2). Blood investigations were done on the first day of admission (Table 1).

**Table 1:** Blood investigations done on the first day of admission

Parameter	Result	Normal range
White blood cells	13,500 (C 10^9^) (/ml)	4000-12000/ul
Neutrophils	61%	-
Lymphocytes	30%	-
Hemoglobin	11.4 (g/dl)	11.0-14.5 g/dl
Platelet count	4.16 (lakhs)	1.5-4.1 lakhs
Prothrombin time	13.20 (seconds)	10.09-13.79 seconds
Partial prothrombin time	36.1 (seconds)	21.76-32.54 seconds
International normalized ratio	1.11	0.85-1.15 (ISI)
Erythrocyte sedimentation rate	11 (mm/h)	Up to 15 mm/hr
C-reactive protein	13.9 (mg/l)	<3.0 mg/l
Procalcitonin	>100 (ng/ml)	<0.08 ng/ml
Total proteins	7.10( gm/dl)	6.4-8.3 gm/dl
Serum urea	20 (mg/dl)	17-49 mg/dl
Serum creatinine	0.42 (mg/dl)	0.19-0.49 mg/dl

Peripheral blood smear examination revealed a normocytic and normochromic picture with mild leukocytosis and no evidence of blasts. Due to the substantial nature of the effusion, an intercostal drainage tube was inserted. The drained pleural fluid, appearing yellowish, thin, and clear, was sent for routine analysis (Table 2).

**Table 2:** Pleural fluid analysis

Parameter	Result	Normal range
Protein	4.7 (gm%)	up to 3 gm%
Glucose	<5 (mg/dl)	>60 mg/dl
Lactate dehydrogenase (LDH)	8123 U/L	143-370 U/L (1 to 15 years of age)
Total cell count	50000 (/mm^3^)	0-150/mm^3^
Neutrophils	60%	-
Leukocytes	30%	-
Adenosine deaminase (ADA)	1293 U/L	40 U/L

In light of the markedly elevated white blood cell (WBC) count in the pleural fluid and the presence of suspicious cells suggestive of blasts, pleural fluid flow-cytometric analysis was conducted. The examination of the pleural fluid smear revealed approximately 65-70% of blasts, measuring 12-14 µm in size, exhibiting a high nuclear-cytoplasmic ratio (N:C ratio), indistinct nucleoli, and scant cytoplasm.

Subsequently, immunophenotyping (flow cytometry) was performed, unveiling 68.64% blasts with dim to moderate CD45 and low side scatter (SSC) on CD45/SSC gating. The blasts were characterized by the expression of cCD3, sCD3, CD4, CD5, CD7, and CD8, while being negative for HLA-DR, CD34, CD117, cMPO, CD10, CD13, CD19, CD20, CD33, CD64, cCD79a, kappa, and lambda light chains. The conclusive impression derived from the analysis indicated involvement by T-cell acute lymphoblastic leukemia (T-ALL). Simultaneously, blood analysis for tumor lysis syndrome (TLS) parameters revealed the following (Table 3).

**Table 3:** Blood analysis for tumor lysis syndrome (TLS)

Parameter	Result	Normal range
Uric acid	20 (mg/dl)	2.4-5.4 mg/dl
Phosphorous	10 (mg/dl)	2.6-4.7 mg/dl
Calcium	4 (mg/dl)	8.8-10.8 mg/dl
Potassium	6.8 (mmol/ml)	3.5-5.1 mmol/ml

Grade 1 changes on the Cairo-Bishop grading scale of TLS were identified, prompting the initiation of rasburicase, followed by allopurinol. Concurrently, hydration and Lasix were administered, leading to a noticeable improvement in the child's condition. The monitoring included tracking the urine output and conducting serial assays of electrolytes and serum uric acid.

**Figure 3:**
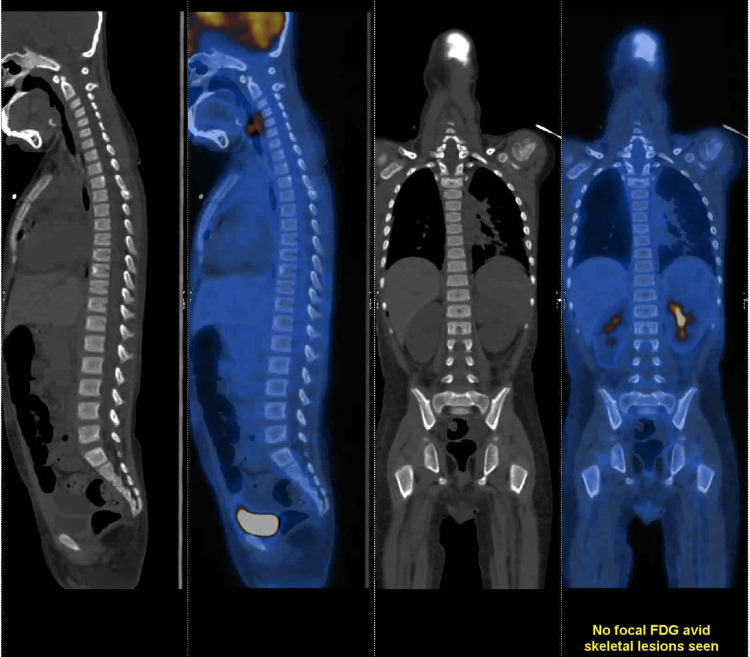
Whole-body PET scan No Fludeoxyglucose F18 (FDG) activity was observed.

Upon confirmation of T-cell lymphoblastic malignancy through flow cytometry, bone marrow and cerebrospinal fluid (CSF) examinations were performed, alongside a whole-body PET-CT scan to assess the extent of involvement. The CSF examination yielded normal results with no evidence of malignant cells on cytology. Bone marrow aspiration indicated an overall hypocellular state with 15-20% blasts, confirmed to be Tdt-positive through immunohistochemistry (IHC) of the bone marrow biopsy. A PET scan (Figure 3), conducted after stabilization and five days of steroid administration, revealed no significant metabolic uptake throughout the body.

The conclusive diagnosis of T-ALL without central nervous system (CNS) involvement was established. The condition was categorized as intermediate risk, prompting the initiation of chemotherapy according to the Berlin-Frankfurt-Munich (BFM)-2002 protocol.

## Discussion

Malignant pleural effusion in pediatric patients is a rare condition, and its prevalence is closely linked to the underlying disease [4]. Parapneumonic effusion or empyema, a complication of pneumonia, is found in 2-12% of pediatric patients with pneumonia and in 28% of children requiring hospitalization [5,6]. It predominantly affects young children, with incidence rates of 3.7 (<two years), 3.9 (two to four years), and 1.3 (five to 17 years) cases per 100,000 populations [5]. The occurrence is gender-neutral. In a study of 61 children with parapneumonic effusion, 11% had an underlying illness or condition [7].

Acute leukemia, primarily ALL, is the most common malignancy in the pediatric age group, constituting 80% of total pediatric neoplasms [8]. Pleural involvement has been reported in ALL, particularly the T-ALL, which often presents with mediastinal mass and pleural effusion. Malignant pleural effusion in hematolymphoid malignancies is most commonly associated with Hodgkin’s lymphoma and non-Hodgkin’s lymphoma, comprising 20-30%.

In pediatric cases of malignant pleural effusion, the presentation typically involves a mass in the lung or mediastinum. However, cases without any mass or clinical features indicating leukemia have not been reported [9]. The presented case adds complexity as the patient initially exhibited febrile illness followed by respiratory symptoms, leading to the initial consideration of an infectious etiology, with empyema being the likely diagnosis. The subsequent diagnosis of malignant pleural effusion was complicated by the patient's presentation with Cairo grade 1 TLS and potential pre-treatment with corticosteroids, contributing to a relatively hypocellular bone marrow and fewer blasts in the biopsy [10].

The final diagnosis of ALL was challenging but eventually confirmed. The patient responded well to the modified ALL-IC BFM 2002 protocol and completed the induction phase successfully. Regular follow-up is ongoing to monitor the patient's progress. The case underscores the complexities in diagnosing malignant pleural effusion in the pediatric population, especially when clinical presentations overlap with other conditions and prior treatments may impact diagnostic findings and prognosis.

## Conclusions

The consideration of leukemia should be considered as a strong differential diagnosis when a child presents with pleural effusion and exhibits a very high cell count in the effusion fluid. It is crucial to avoid the inadvertent administration of corticosteroids before reaching a final diagnosis, as this can potentially interfere with diagnostic accuracy and subsequent management.

In cases where there is any suspicion of malignancy, particularly leukemia, it is advisable to perform a flow cytometric analysis of the pleural fluid. Flow cytometry is a valuable diagnostic tool that can identify and characterize specific cell populations based on their surface markers. This analysis is critical in confirming and providing critical information for the timely and accurate diagnosis of conditions, such as leukemia. This facilitates the initiation of timely and targeted interventions for better patient outcomes.
